# Supplementary material for: Buying Game Derivative Products Is Different from In-Game Purchases: A Mixed-Method Approach
Source: Behav Sci (Basel). 2024 Jul 28;14(8):652. doi: 10.3390/bs14080652 (PMC11351711; doi:10.3390/bs14080652)
Supplement: Supplementary file 1 [file behavsci-14-00652-s001.zip › behavsci-3054446-supplementary.pdf]

SUPPLEMENTARY MATERIALS

Table S1. Demographic profile of the survey participants.

Measure		Frequency	%
Gender	Male	6431	65.2
	Female	2951	29.9
	Secret	482	4.9
Age(years)	≤19	3141	31.8
	20-29	6168	62.5
	≥30	555	5.6
Income	≤2000 RMB	6482	65.7
	2001~5000 RMB	2201	22.3
	5001~10000 RMB	836	8.5
	>10000 RMB	346	3.5
Education	Primary school	20	0.2
	Junior high school	361	3.7
	Senior high school	1528	15.5
	Pursuing or owning a bachelor degree	6822	69.2
	Pursuing or owning a master degree	1133	11.5
Marriage	Unmarried	9557	96.9
	Married	285	2.9

	Other	22	0.2
Job	Full-time job	2023	20.5
	Part-time job	287	2.9
	Student	6862	69.6
	Unemployment	572	5.8
	Other	120	1.2
Religion	None	9465	95.9
	Buddhism	149	1.5
	Taoism	91	0.9
	Christianism	85	0.9
	Islamism	15	0.15
	Other	59	0.60
Years of gaming	≤1 year	4426	44.9
	1~2 years	5306	53.8
	>2 years	132	1.3
Game time/week	≤7 h	4525	45.9
	8~14 h	2680	27.2
	15~21 h	1487	15.1
	22~35 h	873	8.9
	>35 h	299	3.0

Table S2. Demographic profile of the interview participants.

Measure		Frequency	%
Gender	Male	16	72.7
	Female	6	27.3
Age	≤20years old	6	27.3
	20-30years old	16	72.7
Education	High school or lower education	1	4.5
	Associate's degree	2	9.1
	Bachelor's degree	17	77.3
	Postgraduate or higher education	2	9.1
Income	≤1000RMB	5	22.7
	1001~2000RMB	13	59.1
	2001~5000RMB	2	9.1
	5001~10000RMB	1	4.5
	Missing	1	4.5
Marriage	Unmarried	20	90.9
	Missing	2	9.1
Job	Full-time job	1	4.5
	Part-time job	2	9.1
	Student	13	59.1
	Unemployment	4	18.2
	Missing	2	9.1
Religion	None	18	81.9

	Christianism	1	4.5
	Islamism	1	4.5
	Missing	2	9.1
Years of gaming	≤1 year	7	31.8
	1~2 years	12	54.6
	>2 years	1	4.5
	Missing	2	9.1
Game time/week	≤7 h	10	45.5
	8~14 h	3	13.6
	15~21 h	3	13.6
	22~35 h	3	13.6
	>35 h	1	4.5
	Missing	2	9.1

Table S3. Questionnaire for survey.

Factor	Items	References
Character attachment	我和我最喜欢的角色有很强的情绪联结 (I have a strong emotional bond with my favorite game character)	Ko (2021)
	我对我最喜欢的游戏角色有情感上的投入(I feel an emotional investment in my favorite game character)	
	我对我最喜欢的游戏角色饱含深情(I feel very affectionate toward my favorite game character)	
	我对我最喜欢的游戏角色充满热情(I feel very passionate about my favorite game character)	
Problematic gaming	我玩游戏比做其他的事情要用心的多(I am more focused on playing games than doing other activities.)	Pontes et al. (2014)
	我的空闲时间基本上是花在玩游戏上(I basically spend my free time playing games.)	
	我常常因为专心于玩游戏而忽视了身边的许多事(I often neglect many things around me because I'm so focused on playing games.)	
	我在玩完游戏后仍然会沉浸在其中的某些情节里(I find myself immersed in certain aspects of the game even after finishing playing.)	
	玩游戏的时间总是满足不了我的要求(I often think that a whole day is not enough to do everything I need	

	to do in-game.)	
	我花了太多时间玩游戏，以致于影响了自己的学习或工作(I have spent too much time playing games, that it has affected my studies or work.)	
	我曾尝试减少玩游戏的时间，但是没有成功(I've tried to cut down on gaming time, but failed)	
	我常采用上网玩游戏的方式发泄自己的情绪或暂时回避困难(I often use online gaming as a way to vent my emotions or temporarily escape from difficulties.)	Jing et al. (2005)
Impulsive buying tendency	今天有钱就花掉，明天该怎么样就怎么样。(Today, if I have money, I spend it. I don't care about whatever comes my way tomorrow)	
	买任何东西都应慢点来，多多考虑一下。(When buying anything, I should take my time and consider it carefully.)	
	看到喜欢的产品时，有一种不能自禁的感觉。(When I see a product I like, there’s an irresistible feeling.)	
	我会为了改变自己的心情去买一些东西。(Sometimes, I buy things to change my mood.)	
	我不觉得凭借一时冲动而购买有什么风险。(I don’t think there’s any risk in making impulsive purchases.)	
	看重就买是很有趣的事情。(Buying something just	

because you like it is a very interesting thing.)

我买东西都是有计划的。(I always plan my purchases.)

只要喜欢就应该立刻买下来。(If you like it, you should buy it immediately.)

我发现我总是可以抵御住购买的冲动。(I've noticed that I can usually resist the urge to make purchases.)

在我心情不佳的时候我会凭借一时冲动而购买。(When I'm feeling down, I tend to make impulsive purchases.)

有时候我买东西只是为了体验购物的乐趣，而非确实需要。(Sometimes, I buy things just for the joy of shopping, not because I truly need them)

如果看到喜欢的产品，我的眼睛就不愿离开。(When I see a product I like, my eyes can't look away)

买东西的时候最好从各个方面仔细考虑。(It's best to consider various aspects when making a purchase.)

我花钱有点漫不经心。(I'm a bit careless with spending money.)

我总是凭当时的感觉购物。(I usually shop based on my immediate feelings.)

对我来说，购物可以减轻日常生活压力。(For me, shopping can reduce daily life stress)

我的信念是“是要想买就买，不管需不需要”(My belief is ‘if you want to buy it, go ahead, regardless of necessity.’)

喜欢的东西如果不买下来，我总有种不满足的感觉。(If I don’t buy something I like, I feel unsatisfied.)

我买东西没有什么计划性(I don’t plan my purchases)

我经常在没有认识到产品的实际用途时就买下某些产品。(Sometimes, I buy certain products without understanding their practical use.)

我是一个理性的消费者，效用最大化是我的追求目标。(I consider myself a rational consumer, aiming for utility maximization.)

喜欢的东西我希望立即得到它。(When I like something, I hope to get it immediately.)

在购物时，我喜欢先买了再说，以后钱够不够花先不管。(When shopping, I like to buy first and then I don't care if I have enough money to spend later)

做出购物决定时我很少经过太多的思考。(I rarely spend too much time thinking before making a shopping decision.)

多数情况下我都会有计划的购物。(Most of the time, I shop with a plan.)

有时候我在我购物时会遇到“过了这个村就没了这个

	店”的情形，这时我会毫不犹豫地购买。(Sometimes when I am shopping, I will encounter the situations where 'if I don’t buy it now, I’ll miss out', then I will buy without hesitation.)	
Game loyalty	原神总体上令人满意，足以让人在之后继续玩 (Genshin Impact was overall satisfactory enough to reuse later)	Choi (2004)
	当我之后想玩线上游戏时，我会玩原神(I would Genshin Impact when I want to play online games later.)	
In-game purchases	请填写从最开始到现在，您向原神游戏内充值的消费总额有多少，请尽量估算 (Please fill in the total amount you have spent on in-game purchases in Genshin Impact from the beginning up to now. Please provide an estimate if possible. Unit: RMB yuan)	
Derivative products purchases	请填写从最开始到现在，您购买原神周边产品(如人偶、徽章、联动耳机等)的消费总额，请尽量估算 (Please fill in the total amount you have spent on purchasing Genshin Impact peripheral products (such as figurines, badges, collaborative headphones, etc.) from the beginning up to now. Please provide an estimate if possible. (Unit: RMB	

	yuan))
Game frequency	最近一周您游玩原神的频率为一周几天？(How often do you play Genshin Impact in a week recently?)

Table S4. Standardized path coefficients for the hypothesized model.

Path	Estimate	95% confidence interval		*p*
		lower	upper	
WGF→IGP	0.149	0.128	0.171	< 0.001
CA→IGP	0.109	0.088	0.129	< 0.001
PG→IGP	0.020	0.002	0.040	0.033
WGF→DPP	-0.002	-0.019	0.016	0.836
CA→DPP	0.198	0.178	0.217	< 0.001
PG→DPP	-0.014	-0.033	0.006	0.173
WGF→GL	0.195	0.170	0.220	< 0.001
CA→GL	0.267	0.245	0.287	< 0.001
PG→GL	0.004	-0.016	0.024	0.701
GL→IGP	-0.022	-0.042	-0.002	0.032
GL→DPP	0.044	0.023	0.064	< 0.001

Note: WGF = Weekly game frequency; PG = Problematic gaming; CA = Character attachment; DPP = Derivative products purchases (amount, log + 10); IGP = In-game purchases (amount, log + 10); GL = Game loyalty.
